# Gender Equity and Vertically Transmitted Infections: A Country-Level Analysis Across 153 Countries

**DOI:** 10.1089/heq.2020.0097

**Published:** 2021-01-25

**Authors:** Youngmi Lee, Junseok Park, Myeungki Min, Youjin Lee, Youngun Yu, Mi Kyoung Shim, Myeong Gyu Kim

**Affiliations:** ^1^ College of Pharmacy, CHA University, Pocheon, Republic of Korea.; ^2^ College of Pharmacy, Ewha Womans University, Seoul, Republic of Korea.; ^3^ Graduate School of Pharmaceutical Sciences, Ewha Womans University, Seoul, Republic of Korea.

**Keywords:** gender equity, vertical transmission, hepatitis virus, HIV, syphilis

## Abstract

**Purpose:** Gender inequality is a barrier to education toward women and accessibility to health facilities, which are important for preventing vertical transmission. This study was conducted to analyze the impact of gender equity on vertically transmitted infections (hepatitis viruses, human immunodeficiency virus [HIV], and syphilis) using country-level indicators.

**Methods:** The relationship between the Global Gender Gap Index (GGGI), which is indicator of gender equity, and vertical transmission was analyzed. GGGI scores were collected from 153 countries in 2020. Vertical transmission included 10 outcomes for hepatitis viruses, HIV, and syphilis. Generalized linear model (GLM) was used for analyzing the relationship. Other predictors included skilled birth attendant and country income.

**Results:** The median GGGI score was 0.706 (interquartile range, 0.664–0.736). GLM showed that the GGGI score was significantly associated with the incidence of both chronic hepatitis B and C in under 5 years (both *p*<0.001). For HIV, GGGI score was significantly associated with the pregnant women with unknown HIV status (*p*=0.001), no early infant diagnosis (*p*=0.027), and final transmission rate (*p*=0.005). There was no significant predictor for pregnant women who have not received antiretroviral therapy for prevention of mother-to-child transmission. All syphilis indicators have improved in high-income countries compared to low-income countries. GGGI score had a significant association only with no syphilis screening (*p*<0.001).

**Conclusions:** A lower GGGI score was associated with higher vertical transmission of hepatitis and HIV. The improvement of gender equity might prevent vertical transmission of these viruses. Further intervention studies are warranted to verify the results.

## Introduction

Women's empowerment and gender equity are an elemental human right and crucial to achieving wellbeing.^1^ Substantial progress has been accomplished toward women's empowerment and gender equity in accordance with the Millennium Development Goals (MDGs) declaration.^2,3^ Nevertheless, in some parts of the world, women and girls continue to suffer discrimination.^3^ The gender gaps may arise in the following areas: health, educational attainment, economic participation, labor opportunities, political empowerment, and supportive laws and institutions.^4^ The Global Gender Gap Index (GGGI) measures gender gaps in economic participation/opportunity, educational attainment, health/survival, and political empowerment.^4^ The GGGI scores range from 0 to 1, with 0 meaning complete gender inequality and 1 meaning complete gender equity.^4^

Promoting gender equity and empowering women have been known as the key to women's and maternal health.^5^ Women's empowerment may also affect the childrens' and adolescents' health. For this reason, researchers have studied the relationships between health outcomes and gender equity. A systematic review evaluated the impact of gender equity on both women's and men's health across the 48 studies.^6^ In this review, greater gender equity has positive effects on health outcomes such as mental health and self-rated health.^6^ Gender inequality prevented utilization of maternal delivery care and increased maternal mortality ratios.^2,7–10^ In addition, gender inequality was associated with lower prevalence of contraceptive use among adolescents, increased adolescent birth rate, and increased neonatal, infant, and under 5-year mortalities.^11–16^

Vertical transmission, which is known as mother-to-child transmission, influences childrens' health and includes human immunodeficiency virus (HIV) infection, hepatitis B, hepatitis C, and syphilis.^17–19^ Preventing delivery with unknown infections and prevention of mother-to-child transmission (PMTCT) are required to save children and eradicate vertically transmitted infections from the world. To be aware of the necessity of testing and treatment and to be able to receive management during pregnancy are, therefore, important for pregnant women. Gender inequality is a barrier to education toward women and accessibility to health facilities. Few studies have evaluated relationships between country-level gender equity and vertically transmitted infections. Even that focused on the prevalence of infections, especially HIV infection, in women of childbearing age or people who inject drugs rather than on transmission to children.^20–27^ Thus, the aim of this study was to analyze the impact of gender equity on vertically transmitted infections using country-level indicators.

## Methods

IRB approval was waived because national statistics without personal information were used.

### Data

The Global Gender Gap Report 2020 reported GGGI scores and its subindices.^28^ This study included 153 countries where the GGGI score was reported in 2020.^28^ Country income and skilled birth attendant (SBA), which could contribute to maternal and child health, were investigated as confounding factors. Country income, gross national income (GNI) per capita in 2019, which was calculated using World Bank Atlas method, was collected from the World Bank database.^29^ Country income, which is a continuous variable, was converted into a categorical variable. Country income was classified into four groups: GNI per capita of >12,536 USD for high income (HI); 4046–12,535 USD for upper-middle income (UMI); 1036–4045 USD for lower-middle income; and ≤1035 USD for low income (LI).^30^ SBA refers to the percentage of births attended by skilled health personnel and the most recent value was used.^31^

### Outcomes

In this study, 10 vertical transmission outcomes for hepatitis viruses, HIV, and syphilis in 2017 were evaluated.

For hepatitis viruses, incidence of chronic hepatitis B and that of hepatitis C in under 5-year old (cases per 100,000) were investigated because the risk of acquiring chronic hepatitis is greatest in the perinatal period, with up to 90% of infected newborns becoming chronic carriers.^18,32^

Four outcomes for HIV were selected: pregnant women with unknown HIV status (%), pregnant women who have not received antiretroviral therapy (ART) for PMTCT (%), no early infant diagnosis defined as testing within 2 months of birth (%), and final transmission rate, including breastfeeding period.^33^

For syphilis, maternal syphilis prevalence (%), no syphilis screening (%), no syphilis treatment (%), and congenital syphilis rate (cases per 100,000 live births) were investigated.^34^

Some percentage outcomes are contrary to those reported in the literature. The data for those outcomes were taken from 100 minus the percentage value reported in the literature.

### Statistical analysis

The data did not meet the assumption of a multiple linear regression that the residuals are normally distributed. Alternatively, a generalized linear model (GLM), which allows for considerable flexibility, was used for analyzing the relationship between the set of predictors and response variable.^35^ Predictors included GGGI score or subindices, SBA, and country income. GGGI and subindex were exclusively included in the model, taking into account multicollinearity. LI group was used as the reference in the GLM analysis.

The distribution of the response variable, Yi, was described as gamma or inverse-Gaussian families of distribution, which were suitable for the right-skewed response variable. The GLM is further specified by the link function to linearize the relationship.^35^ Various link functions such as identity, log, inverse, inverse square, and square root were explored.^35^ The model with the minimum Akaike's Information Criterion (AIC) or Bayesian Information Criterion (BIC) was selected as the best fitting model.^36,37^

An alpha of 0.05 was used as the cutoff for significance. SPSS version 21 (IBM Corp., Armonk, NY) was used for statistical analysis.

## Results

### Characteristics of study countries

Characteristics of study countries are shown in Table 1. The median GGGI scores for 153 countries was 0.706 (interquartile range [IQR], 0.664–0.736). The median score for educational attainment and health/survival among the GGGI subindices was close to 1 (0.992 [IQR, 0.956–0.999] and 0.974 [IQR, 0.969–0.979], respectively).

**Table 1.** Characteristics of Study Countries

Characteristics	Countries (*n*=153)
GGGI score^a^	0.706 (0.664–0.736)
Economic participation/opportunity^a^	0.673 (0.598–0.736)
Educational attainment^a^	0.992 (0.956–0.999)
Health/survival^a^	0.974 (0.969–0.979)
Political empowerment^a^	0.189 (0.111–0.278)
SBA (%)^a^	97.95 (81.55–99.7)
GNI per capita, Atlas method (USD)^a^	6040 (2220–19,320)
HI^b^	53 (34.6)
UMI^b^	40 (26.1)
LMI^b^	42 (27.5)
LI^b^	18 (11.8)
Incidence of chronic hepatitis B (cases/100,000)^a^	0.5 (0.1–0.8)
Incidence of chronic hepatitis C (cases/100,000)^a^	0.3 (0.2–0.7)
Pregnant women with unknown HIV status (%)^a^ (*n*=81)	16 (5–52)
Pregnant women who have not received ART for PMTCT (%)^a^ (*n*=81)	27 (15–60)
No early infants diagnosis of HIV (%)^a^ (*n*=94)	49 (30–76)
Final transmission rate of HIV^a^ (*n*=79)	15 (10–23)
Maternal syphilis prevalence (%)^a^	0.2 (0.06–1.1)
No syphilis screening (%)^a^	10 (3–38)
No syphilis treatment (%)^a^	7 (5–16)
Congenital syphilis rate (cases/100,000 live births)^a^	70 (8–519)

^a^ Median (interquartile range).

^b^ *n* (%).

ART, antiretroviral therapy; GGGI, Global Gender Gap Index; GNI, gross national income; HI, high income; HIV, human immunodeficiency virus; LI, low income; LMI, lower-middle income; PMTCT, prevention of mother-to-child transmission; SBA, skilled birth attendant; UMI, upper-middle income.

### Relationship between GGGI and vertical transmission outcomes

GGGI score was significantly related to both hepatitis outcomes, three of the four HIV outcomes, and one syphilis outcome. For all outcomes, using GGGI rather than using GGGI's subindices has built GLM models that better describe the outcomes.

### Hepatitis virus

The median incidence of chronic hepatitis B and that of hepatitis C in under 5 years was 0.5 (IQR, 0.1–0.8) and 0.3 (IQR, 0.2–0.7) cases per 100,000, respectively (Table 1).

GLM with gamma family and square root link was selected as the best-fit model for the incidence of chronic hepatitis B. As the GGGI score increased, that is, as the gender gap decreased, the incidence of chronic hepatitis B decreased significantly (*p*<0.001; Table 2). GLM with inverse family and log link was used to describe the incidence of chronic hepatitis C. GGGI score was also found to be a significant predictor of the incidence of chronic hepatitis C (*p*<0.001; Table 2).

**Table 2.** Generalized Linear Models for Outcomes of Hepatitis Virus

GLM	*n*	Predictors	β	95% CI
Incidence of chronic hepatitis B (cases/100,000)				
μ=(β_0_ + β_1_×GGGI + β_2_×SBA + β_3_×country income)^2^ (Gamma family, square root link)	134	Intercept (β_0_)	2.83	2.26 to 3.40
		GGGI (β_1_)	−3.38	−4.18 to −2.58^*^
		SBA (β_2_)	0.004	0.0003 to 0.007^*^
		LI (β_3_)	0	Reference
		LMI (β_3_)	−0.032	−0.196 to 0.132
		UMI (β_3_)	−0.081	−0.288 to 0.127
		HI (β_3_)	−0.028	−0.239 to 0.183
Incidence of chronic hepatitis C (cases/100,000)				
μ=exp(β_0_ + β_1_×GGGI + β_2_×SBA + β_3_×country income) (Inverse family, log link)	148	Intercept (β_0_)	3.54	1.79 to 5.28
		GGGI (β_1_)	−7.65	−9.98 to −5.32^*^
		SBA (β_2_)	0.018	0.008 to 0.029^*^
		LI (β_3_)	0	Reference
		LMI (β_3_)	−0.425	−1.042 to 0.191
		UMI (β_3_)	−0.566	−1.315 to 0.183
		HI (β_3_)	−0.576	−1.325 to 0.173

^*^ *p*<0.05.

GLM, generalized linear model.

### Human immunodeficiency virus

Information related to HIV was reported mainly in low- and middle-income countries, while HI countries were omitted (Table 1). The percentage of pregnant women with unknown HIV status and who have not received ART for PMTCT was 16% (IQR, 5–12) and 27% (IQR, 15–60), respectively. Infants with a median value of 49% (IQR, 30–76) were not diagnosed early, and the median of the final transmission rate was 15 (IQR, 10–23).

GLM with inverse family and identity link best fitted data on pregnant women with unknown HIV status. GGGI was significantly associated with the pregnant women with unknown HIV status (*p*=0.001; Table 3). GLM for pregnant women who have not received ART for PMTCT showed that none of the evaluated predictors was significant (Table 3).

**Table 3.** Generalized Linear Models for Outcomes of Human Immunodeficiency Virus

GLM	*n*	Predictors	β	95% CI
Pregnant women with unknown HIV status (%)				
μ=β_0_+β_1_×GGGI + β_2_×SBA + β_3_×country income (Inverse family, identity link)	72	Intercept (β_0_)	190.7	92.0 to 289.5
		GGGI (β_1_)	−198.5	−318.1 to −78.8^*^
		SBA (β_2_)	−0.28	−0.74 to 0.19
		LI (β_3_)	0	Reference
		LMI (β_3_)	−1.15	−12.1 to 9.82
		UMI (β_3_)	6.36	−8.98 to 21.7
		HI (β_3_)	0.58	−38.1 to 39.3
Pregnant women who have not received ART for PMTCT (%)				
μ=(β_0_ + β_1_×GGGI + β_2_×SBA + β_3_×country income)^2^ (Inverse family, square root link)	72	Intercept (β_0_)	13.8	5.7 to 21.8
		GGGI (β_1_)	−10.2	−21.5 to 1.1
		SBA (β_2_)	−0.03	−0.07 to 0.02
		LI (β_3_)	0	Reference
		LMI (β_3_)	1.42	−0.41 to 3.25
		UMI (β_3_)	1.05	−0.84 to 2.94
		HI (β_3_)	−1.43	−3.14 to 0.27
No early infant diagnosis (%)				
μ=β_0_ + β_1_×GGGI + β_2_×SBA + β_3_×country income (Gamma family, identity link)	59	Intercept (β_0_)	191.3	100.4 to 282.2
		GGGI (β_1_)	−148.1	−279.4 to −16.7^*^
		SBA (β_2_)	−0.54	−1.12 to 0.04
		LI (β_3_)	0	Reference
		LMI (β_3_)	6.28	−15.2 to 27.8
		UMI (β_3_)	−0.94	−24.5 to 22.7
		HI (β_3_)	−14.4	−50.8 to 22.1
Final transmission rate				
μ=β_0_ + β_1_×GGGI + β_2_×SBA + β_3_×country income (Gamma family, identity link)	74	Intercept (β_0_)	66.8	42.8 to 90.8
		GGGI (β_1_)	−50.7	−86.1 to −15.4^*^
		SBA (β_2_)	−0.22	−0.38 to −0.06^*^
		LI (β_3_)	0	Reference
		LMI (β_3_)	5.06	−0.14 to 10.25
		UMI (β_3_)	2.01	−3.55 to 7.57
		HI (β_3_)	−2.56	−10.83 to 5.72

^*^ *p*<0.05.

GLM with gamma family and identity link was best suited for both no early infant diagnosis and final transmission rate. The high GGGI scores were significantly associated with a decrease in both outcomes (*p*=0.027 and *p*=0.005, respectively; Table 3). In addition, as the percentage of SBA increased, the final transmission rate significantly decreased (*p*=0.006; Table 3).

### Syphilis

Maternal syphilis prevalence was 0.2% (IQR, 0.06–1.1). Both the percentage of no screening and no treatment were low (10% [IQR, 3–38] and 7% [IQR, 5–16], respectively). Congenital syphilis rate was 70 (IQR, 8–519) cases/100,000 (Table 1).

GLM with gamma family was used for four syphilis outcomes. The prevalence of maternal syphilis was significantly lower in HI countries than in LI countries (*p*=0.005; Table 4). Country income was a significant predictor of no syphilis screening and no syphilis treatment, and both ratios were significantly lower than those of LI countries in HI countries (*p*=0.003 and *p*=0.019, respectively; Table 4). Increasing GGGI scores and increasing SBA percentages also significantly reduced the ratio of no syphilis screening (*p*<0.001 and *p*=0.09, respectively; Table 4).

**Table 4.** Generalized Linear Models for Outcomes of Syphilis

GLM	*n*	Predictors	β	95% CI
Maternal syphilis prevalence (%)				
μ=exp(β_0_ + β_1_×GGGI + β_2_×SBA + β_3_×country income) (Gamma family, log link)	151	Intercept (β_0_)	3.42	−0.09 to 6.93
		GGGI (β_1_)	−2.34	−7.23 to 2.55
		SBA (β_2_)	−0.02	−0.04 to 0.004
		LI (β_3_)	0	Reference
		LMI (β_3_)	−0.45	−1.24 to 0.33
		UMI (β_3_)	−0.64	−1.60 to 0.32
		HI (β_3_)	−1.37	−2.33 to −0.41^*^
No syphilis screening (%)				
μ=(β_0_ + β_1_×GGGI + β_2_×SBA + β_3_×country income)^2^ (Gamma family, square root link)	141	Intercept (β_0_)	16.8	12.5 to 21.2
		GGGI (β_1_)	−7.52	−11.4 to −3.62^*^
		SBA (β_2_)	−0.06	−0.10 to −0.02^*^
		LI (β_3_)	0	Reference
		LMI (β_3_)	−1.29	−3.01 to 0.43
		UMI (β_3_)	−1.81	−3.74 to 0.13
		HI (β_3_)	−2.93	−4.84 to −1.01^*^
No syphilis treatment (%)				
μ=β_0_ + β_1_×GGGI + β_2_×SBA + β_3_×country income (Gamma family, identity link)	121	Intercept (β_0_)	27.4	3.25 to 51.4
		GGGI (β_1_)	2.00	−23.5 to 27.5
		SBA (β_2_)	−0.10	−0.33 to 0.13
		LI (β_3_)	0	Reference
		LMI (β_3_)	1.57	−8.29 to 11.4
		UMI (β_3_)	−0.86	−11.7 to 9.95
		HI (β_3_)	−12.8	−23.5 to −2.11^*^
Congenital syphilis rate (cases per 100,000 live births)				
μ=(β_0_ + β_1_×GGGI + β_2_×SBA + β_3_×country income)^−1^ (Gamma family, inverse link)	151	Intercept (β_0_)	0	−0.004 to 0.004
		GGGI (β_1_)	0	−0.006 to 0.006
		SBA (β_2_)	0	−0.00001 to 0.00002
		LI (β_3_)	0	Reference
		LMI (β_3_)	0.001	−0.0004 to 0.002
		UMI (β_3_)	0.003	0.001 to 0.005^*^
		HI (β_3_)	0.009	0.005 to 0.014^*^

^*^ *p*<0.05.

Congenital syphilis rate was associated with country income. There has been a significant decrease in congenital syphilis rates in UMI and HI countries compared to LI countries (*p*=0.01 and *p*<0.001, respectively; Table 4).

## Discussion

This study investigated the association between gender equity and vertically transmitted infections at country level. The GGGI, an indicator of gender equity, was related with several outcomes of vertically transmitted infections. The high GGGI score, which means a decrease in the gender gap, was related to the improvement of vertical infection outcomes. The significant influence of GGGI was derived from an analysis that reflected SBA and country income, which are thought to affect vertical infections.

Vertical transmisson of hepatitis B virus depends on the maternal hepatitis B e antigen (HBeAg) status, which is approximately 10–30% in HBeAg(−) carriers and considerably increases to 90% in HBeAg(+).^18^ Hepatitis B immunoglobulin administered to infants at birth reduced mother-to-child transmission from 21% to 6% in HBeAg(+) carriers and 2.6% to 1% in HBeAg(−) carriers.^38^ Pregnant hepatitis B carriers can also benefit from receiving antivirals for PMTCT if they recognize their conditions.^18^ Mother-to-child transmission of hepatitis C virus is the leading cause of pediatric hepatitis C and can occur during intrauterine and peripartum period.^39,40^ Maternal ribonucleic acid (RNA) viral load >6 log IU/mL was identified as an important determinant of vertical transmission.^39^ Currently, there is no proven safe and effective approach for the PMTCT of hepatitis C virus.^40,41^ Nevertheless, awareness of hepatitis C is expected to reduce the vertical propagation of hepatitis C. In particular, unlike HIV that develops severe symptoms, hepatitis virus carriers may not know their status. Self-awareness of viral hepatitis status was significantly lower in people with less education.^42^ Thus, a high GGGI score means improvement in women's educational attainment and would have been associated with a decrease in the incidence of chronic hepatitis B and C in under 5 years in this study.

HIV is one of the world's most serious health challenges. Fortunately, antiviral treatment has benefited from preventing the virus from mother-to-child transmission.^43,44^ However, gender inequality restricted pregnant women from knowing their HIV status, and infants from receiving a virologic test within 2 months of birth in this study. For this reason, the final transmission rate would have increased with lower GGGI scores. Women with decision-making power tended to have a higher likelihood of preventing vertical infection of HIV.^27^ Also, gender inequality may make it impossible to negotiate with partners infected with HIV or use of condoms.^45^ Similarly gender inequality has been reported to be linked to under-five mortality due to HIV transmission in another study.^11^

Syphilis vertical transmission caused congenital syphilis and neonatal deaths in 76.8% of untreated pregnant patients.^46^ Congenital syphilis remains the most common vertically transmitted infection, which was more frequently reported than perinatal HIV infections.^46,47^ However, latent syphilis is hard to be aware of due to the absence of clinical symptoms, which continues to cause mother-to-child transmission of syphilis.^46^ Early initiation of antenatal care and antenatal syphilis diagnosis could prevent adverse pregnancy outcomes.^48^ For this reason, recognition, appropriate maternal testing, and treatment during the pregnancy have been suggested.^46,47^ Gender equity improved syphilis screening coverage during pregnancy and might improve awareness of syphilis. Unlike the hypothesis, however, the gender gap had no significant association with the rest of the syphilis indicators. Instead, country income was significantly linked to maternal syphilis prevalence, syphilis screening coverage, syphilis treatment coverage, and congenital syphilis rate.

Besides gender equity, SBA was associated with final transmission rate of HIV and syphilis screening. SBA is a key indicator in measuring progression toward advanced women's health.^49^ In a previous study, countries with the highest SBA had significant reduction in neonatal mortality.^50^ Furthermore, reduction in accessibility to antenatal care and SBA limits the chances to provide PMTCT services.^49,51^ Therefore, high SBA would have had a positive effect on HIV and syphilis outcomes.

This study has several limitations. First, this country-level study ignored personal factors and consequences. There is no information on maternal infectious status and receiving PMTCT at individual level, which are important factors of vertically transmitted infections. Second, the year in which each of the indicators was investigated, including the GGGI data, is not completely the same. Finally, there may be some confounding factors that were not sufficiently considered in this study.

## Conclusion

A lower GGGI score was associated with higher vertical transmission of hepatitis and HIV. Thus, the improvement of gender equity might prevent the mother-to-child transmission and further contribute to the eradication of these viruses. From a public health perspective, policy support will be needed to reduce gender gaps. It would be worthwhile to elucidate the underpinning mechanisms or verify the results of this study through future intervention studies that reduce the gender gap at the individual level.

## Abbreviations Used

AIC: Akaike's Information Criterion
ART: antiretroviral therapy
BIC: Bayesian Information Criterion
GGGI: Global Gender Gap Index
GLM: generalized linear model
GNI: gross national income
HBeAg: hepatitis B e antigen
HI: high income
HIV: human immunodeficiency virus
IQR: interquartile range
LI: low income
MDGs: Millennium Development Goals
PMTCT: prevention of mother-to-child transmission
RNA: ribonucleic acid
SBA: skilled birth attendant
UMI: upper-middle income
